# Effects of probiotics on growth, survival, and intestinal and liver morphometry of Gangetic mystus (*Mystus cavasius*)

**DOI:** 10.1016/j.sjbs.2023.103683

**Published:** 2023-05-12

**Authors:** Md Abdul Gaffar, Md Kausar Zaman, Md Sameul Islam, Muallimul Islam, Md Kabir Hossain, Sheik Istiak Md Shahriar, Md Shahjahan

**Affiliations:** Laboratory of Fish Ecophysiology, Department of Fisheries Management, Bangladesh Agricultural University, Mymensingh 2202, Bangladesh

**Keywords:** Aquaculture, Catfish, Growth, Immunity, Probiotics

## Abstract

The usage of probiotics proved advantageous in aquaculture due to its positive impact on fish growth, immune response and environment. This study was aimed to assess the effects of probiotics on growth, survival and histometry of intestine and liver in Gangetic mystus (*Mystus cavasius*) using two separate experiments for a period of 8 weeks (in aquaria) and 16 weeks (in earthen ponds). Three different probiotic treatments were incorporated i.e. commercial probiotic one; CP-1 (T1), commercial probiotic two; CP-2 (T2), Lab developed (Lab dev.) probiotic (T3) including a control. The results indicated that the probiotics usage especially Lab dev. probiotic (T3) significantly improved the growth parameters such as weight gain (g) and specific growth rate (SGR, %/day) as well as ensured better feed conversion efficiency. Zero mortality was observed in aquaria whereas probiotic application enhanced survivability in earthen ponds. Moreover, all probiotic treatment exhibited positive results for different histo-morphometric features of intestine and liver. Mucus secreting goblet cells and fattening of mucosal fold increased significantly with probiotic usage. The amount of regular shaped nucleus was maximum in T3 with least intra cellular distance between liver tissues in earthen ponds. The greatest value for hemoglobin with lowest glucose level was observed in T3 as well. Furthermore, probiotic ensured low concentration of ammonia during culture. Overall, it was anticipated that the application of probiotics in Gangetic mystus culture resulted positive effect on its growth, feed utilization, survivability, histo-morphometry, immunity and hematological parameters.

## 1 Introduction

Aquaculture is one of the rapidly evolved promising food providing sectors in the world; income generating source; creating employment opportunities; consistently providing animal protein and ensuring food security (FAO, 2019; Subasinghe et al., 2009). With the advancement of technology, traditional fish farming has been shifted toward modern aquaculture operations with the purpose to obtain more yields (Akter et al., 2021, Mohapatra et al., 2013). Consequently, poor growth, additional feed cost, pathogen invasion, outbreak of fatal diseases and water quality deterioration are associated problems emerged with the surplus productivity in intensified aquaculture system (EL-Haroun et al., 2006, Liu et al., 2012, Mohammadi et al., 2021, Tan et al., 2016). According to Nguyen et al. (2020) the profit generated from aquaculture operation largely relies on the physical quality of feed, nutritional profile and its cost. Besides that ability of fish towards better feed utilization, their intestinal and liver functioning and immunity against stress is directly reflected in growth and overall productivity (Dawood, 2021, Hossain et al., 2022a, Martin and Król, 2017).

Application of probiotics in aqua-farming is one of the modern advancement that could provide breakthrough since it has potency to be used as nutritional supplement, enhances beneficial micro-biota that take care of intestine and liver health, increases feed intake efficiency, escalates growth, balances water quality parameters, improves immune system to fight against pathogen (El-Saadony et al., 2021, Hai, 2015, Martínez Cruz et al., 2012). Probiotics are the assemblages of beneficial living microorganisms mostly comprising bacteria and/or yeast that inhabit and colonize in the gastrointestinal tract of host organisms, bolster the diversity of existing gut micro-biome and subsequently convey health benefit through eliminating, replacing or suppressing harmful microorganisms (Amoah et al., 2019, Rohani et al., 2022). Furthermore, according to Ringø, (2020), application of probiotics in aquaculture is evident in enhancing growth performance through ensuring better digestion with the proliferation of useful microbes’ colony in intestine that provide favorable condition to facilitate enzymatic activity. Besides, probiotics have the efficiency to improve intestinal morphology and liver functioning that modulating immune response and hematological parameters of host species; resulting increased survival and disease resistance (Ding et al., 2021, Hossain et al., 2022a). Incorporation of probiotics in culture media or with supplementary diet provides additional nutrition and their secreted enzyme escalates the feed utilization that ultimately reduces feed cost (Martínez Cruz et al., 2012). Study reported that probiotic can contribute 20% of the total amount of feed required for fishes (De Schryver et al., 2008). In addition, probiotic helps to maintain water quality parameters within optimum range especially balances nitrate and ammonia as well as reduces environmental stress (Mujeeb Rahiman et al., 2010, Wang et al., 2008).

Nevertheless, according to Hai (2015), a diverse range of gram positive bacteria genus is commonly used in fish and shellfish culture such as *Bacillus* spp., *Lactobacillus* spp., *Lactococcus* spp., *Carnobacterium* spp., *Rhodobacter* spp., *Enterococcus* spp., and *Micrococcus* spp. etc. Though any single strain of these bacteria brings productivity in culture, integration of two or more strains provide better performance (Hossain et al., 2022b). For example, the combination of *Bacillus* and *Lactobacillus* are extensively used in aquaculture since they provide multiple benefits. They promote growth parameters of aquatic organisms such as weight gain, SGR, viscera somatic index (VSI), hepato somatic index (HSI), FCR and survival (Apún-Molina et al., 2009, Mujeeb Rahiman et al., 2010, Silva et al., 2021, Wang and Gu, 2010); improved immunity or disease resistance (Aly et al., 2008, Mohammadi et al., 2021, Panigrahi et al., 2007, Ridha and Azad, 2012); and ensured better gut morphology (Standen et al., 2015) in different fishes and shellfishes. Besides that Moriarty (1998) used commercial probiotic containing *Bacillus* sp. and recorded increased shrimp viability in ponds. Another supplementation of commercial probiotics named ‘Biogen’ containing *B. subtilis* spp., *Allicin* and enzymes in tilapia (*Orechromis niloticus*) culture anticipated enhanced growth and feed utilization (EL-Haroun et al., 2006). Standen et al. (2016) recorded that the application of commercial probiotic imparted positive results for growth and immunity for Nile tilapia. Queiroz and Boyd (1998) used ‘Biostart’ to improve growth and water quality of channel catfish ponds.

Gangetic mystus (*Mystus cavasius*) is a freshwater catfish species extensively distributed in earthen ponds, canals, swamps, rivers, tributaries of South-East Asian countries (Talwar and Jhingran, 1991; Tripathi, 1996, Rahman et al., 2004). This omnivorous fish species has become popular commodity among consumers due to its finest taste and nutritional profile such as high protein content, low fat percentage and considerably rich phosphorus and calcium content (Hossain et al., 1999, Rahman, 1999). Moreover, this species has been considered as potential aquaculture species due to its culture potential in high stocking density, short life cycle, good feed conversion efficiency, faster growth and high market demand (Rahman et al., 2021). Lately, the intensification of Gangetic mystus culture led to poor growth and increasing susceptibility to disease, thus, farmers often failed to get expected profit because of reduced productivity (Hosen et al., 2017, Uddin et al., 2020). Since, probiotic is evident to improve wellbeing of catfishes through promoting growth, nutrient digestibility, and survivability (Bunnoy et al., 2019, Queiroz and Boyd, 1998); improving intestinal morphology and immunity (Boonanuntanasarn et al., 2019, El Euony et al., 2020); hematology (Dahiya et al., 2012); and water quality parameters (Al-Dohail et al., 2011, Thurlow et al., 2019) and as there is no such record yet established for Gangetic mystus, therefore, this research was conducted with an aim to assess the effects of probiotics on growth, feed efficiency, survivability, intestinal and liver morphology as well as hematological parameters of Gangetic mystus (*Mystus cavasius*).

## 2 Materials and methods

### 2.1 Experimental design

A total of 8240 active and healthy fries having an average weight of 0.73 ± 0.08 g were collected from a local hatchery named Sharnalata Agro-Fisheries Ltd., Mymensingh, Bangladesh (24.61°N, 90.34°E). The collected fries were subdivided in order to carry two separate experiments. Two commercial probiotics (CP-1, Bangladesh and CP-2, China), a Laboratory developed (Lab dev,) probiotic (Bangladesh) and a control (no probiotic) were incorporated to conduct these experiments. The composition of commercial probiotics (according to manufacturer) was such; CP-1 with *Bacillus subtillis*, *B. polymyxa*, *B. licheniformis*, *B. pumilus*, *B. coagulans*, *B. amyloliquefaciens*, *B. megaterium*, *Aspergillus niger*, *A. oryzae* and CP-2 with *B. subtillis, B. licheniformis, B. pumilus, B. megaterium, Rhodococcus* spp.*, Rhodobacter* spp.*, Nitrosomonus and Nitrobacter.* The Lab dev. probiotic mainly consists of *B. subtilis, L. buchneri* and *L. plantarum* with a colony forming unit (CFU) of 1x10^9^ and 1x10^11^ per ml; respectively.

### 2.2 First and second experiments

The first experiment was conducted inside glass aquaria. It had three treatments (i.e. CP-1, T1; CP-2, T2; Lab dev., T3) including a control and each treatment had three replicates. In total, 12 glass aquaria (dimension, 75x45x45 cm; water holding capacity, 40 L) were used and in each aquarium, 20 fish were stocked. After stocking the fish, the aquaria were kept at natural photoperiod for about 2 weeks before applying probiotic in the aquaria. The second experiment was conducted in four earthen ponds (area, 2 decimals) stocking 1000 Gulsha fries per decimal for 16 weeks using the same treatments of first experiment. Commercial probiotics were activated using molasses according to the manual of manufacturer. Subsequently, 1 ml liquid probiotics were inoculated to culture unit per liter of water initially and 1 ml per liter in every alternative day was incorporated to maintain 3.5% (35 ml) in Imhoff cone test. The fishes were fed with a commercial feed (0.8 mm floating pellet feed, Quality feeds limited, Bangladesh) containing 38% protein and given fishes at the rate of 5% of body weight daily for twice time (9:00 am and 5:00 pm). The amount of feed required for fishes was adjusted quarterly.

#### 2.2.1 Estimation of growth, feed efficiency and survivability

During experiment, fishes lied on bottom with no swimming and opercula movement were considered dead. At the end of experiments, final body weight (FBW) of each fish was measured in gram (g). Growth was calculated using the equations; weight gain (WG) = (final weight (g) – initial weight (g)), specific growth rate (SGR %/day) = (ln final wt (g) – ln initial wt (g)/ no. of rearing days)*100, viscera somatic index (VSI) = (viscera wt/ body wt)*100 and hepatosomatic index (HSI) = (liver wt/ body wt)*100. Feed utilization was assessed using feed conversion ratio (FCR) = dry feed fed (g)/ live wt gain (g) and survivability was estimated as SR (%) = (final no. of fishes/ initial no. of fishes)*100.

#### 2.2.2 Estimation of intestinal histo-morphometry

Five (05) fishes from each replicate of first experiment were dissected. According to the process followed by Bullerwell et al. (2016), the intestine was removed carefully and preserved in marked vials containing Bouin’s fluid for 24 h; later on transferred to 70% alcohol for storage and kept at 4°C for further processing. Tissue processing initiated with fixation in one hour formalin treatment and dehydration was done in a series of alcohol graduation to remain the cellular component intact. Infiltration and embedment was followed in molten paraffin wax. Subsequently, processed tissue was trimmed in 5 µm thickness in microtome machine. Tissue sections were placed on glass slides and air dried overnight. Xylene treatments were followed to remove wax and a series of descending alcohol treatments were performed. After staining with Hematoxylin-Eosin tissue sections were investigated under electronic microscope (MCX100, Micros; Austria) mounted with camera (AmScope MA 1000). Changes in intestinal histo-morphometry were determined measuring villus length (µm), villus width (µm), villus area (mm^2^), crypt depth (µm), intestinal wall and muscle thickness (µm). Immune indicators such as number of goblet cells, lamina propia width (µm), fattening of mucosal fold (µm) and enterocyte width (µm) were estimated. Livers were separated from five fishes (05) in second experiment and tissue were processed to measure histo-morphometry i.e. the shape of nucleus (whether regular or irregular) and distance between tissues (µm).

#### 2.2.3 Estimation of hematometric indices (second experiment)

Blood samples of five (05) fish were collected from each treatment. Micropipette was used to collect blood samples from the caudal vein in order to estimate hemoglobin (Hb; g/dL) and glucose level (Glu; mg/dL). These two parameters were measured directly with digital meter using separate glucose and hemoglobin strips (EasyMate; Glu/Hb double monitoring system, Biotic, Taiwan; model ET232).

### 2.3 Water quality parameters

Four important water quality parameters such as water temperature (°C), dissolved oxygen (mg/l), pH and ammonia (mg/l) were measured biweekly between 8.30 and 9.30 am using digital Celsius thermometer, portable dissolved oxygen meter (Lutron, model no.: DO-5509), portable pH meter (HI 98129, Hanna Ins.) and API ammonia test kit, respectively for both experiments.

### 2.4 Data analysis

Normality and homogeneity test were done to observe distribution and variability of data before analysis in SPSS ver. 18.0 (IBM, Chicago, USA). The mean, standard deviation (SD), and one way analysis of variance (ANOVA) among treatments at 5% significance interval also carried out. Principal component analysis (PCA) was done in Minitab statistical software ver. 20.

## 3 Results

### 3.1 Growth, feed efficiency and survivability (first experiment)

The findings of final body weight (g), weight gain (g), specific growth rate (SGR, %/day), viscerosomatic index (VSI), feed conversion ratio (FCR) and survivability (SR, %) of the Gangetic mystus reared with probiotics in aquarium is presented in Table 1. One way ANOVA showed that initial body weight was not significantly different (p > 0.05) among treatments but final body weight (g), SGR (%/day), VSI, FCR, and SR (%) were significantly different (p < 0.05). The highest final body weight (g) was observed in T3 and the lowest final body weight (g) was observed in control. Similar results were observed in case of weight gain (g) and SGR (%/day). Treatments with probiotic showed better VSI and FCR compared to control. Zero mortality was recorded during the experiment (Table 1). .

**Table 1:** Growth, feed utilization and survival of the Gangetic mystus reared with probiotics in aquarium for 8 weeks.

Parameters	Probiotics			
	Control	CP-1 (T1)	CP-2 (T2)	Lab dev. (T3)
Initial BW (g)	0.72 ± 0.10^a^	0.73 ± 0.09^a^	0.73 ± 0.09^a^	0.72 ± 0.10^a^
Final BW (g)	2.78 ± 0.40^a^	3.25 ± 0.41^ab^	3.84 ± 0.79^b^	3.88 ± 0.50^b^
Weight gain (g)	2.07 ± 0.33^a^	2.52 ± 0.38^ab^	3.12 ± 0.74^b^	3.43 ± 0.47^b^
SGR (%)	1.05 ± 0.07^a^	1.16 ± 0.10^ab^	1.28 ± 0.13^b^	1.34 ± 0.08^b^
VSI	2.94 ± 0.57^b^	2.61 ± 0.31^ab^	2.51 ± 0.67^ab^	2.47 ± 0.64^a^
FCR	1.98 ± 0.35^b^	1.61 ± 0.23^ab^	1.35 ± 0.32^a^	1.18 ± 0.15^a^
SR (%)	100 ± 0.00	100 ± 0.00	100 ± 0.00	100 ± 0.00

BW; body weight, SGR; specific growth rate, VSI; viscerosomatic index; FCR; feed conversion ratio, SR; survival rate. Values with different alphabetical superscripts in a row differ significantly (p < 0.05) among different probiotics treatments. All values are expressed as mean ± SD.

### 3.2 Growth, feed efficiency and survivability (second experiment)

The results of final body weight (g), weight gain (g), SGR (%/day), hepatosomatic index (HSI), VSI, FCR and survivability (%) of the Gangetic mystus reared with probiotics in pond is presented in Table 2. The highest final body weight (g), weight gain (g) and SGR (%/day) was observed in T3. Comparatively better FCR was also observed in Lab dev. probiotic (T3). One way ANOVA showed that T3 was significantly different (p < 0.05) in terms of weight gain (g) and FCR. The value of HSI was reported greatest in CP-2 and had no significant difference with other treatments (p > 0.05). Survivability (%) was improved positively with the application of probiotics.

**Table 2:** Growth, feed utilization and survival of the Gangetic mystus (*Mystus cavasius*) reared with probiotics in aquarium for16 weeks.

Parameters	Probiotics			
	Control	CP-1 (T1)	CP-2 (T2)	Lab dev. (T3)
Initial BW (g)	0.73 ± 0.09	0.74 ± 0.08	0.73 ± 0.08	0.73 ± 0.09
Final BW (g)	10.99 ± 1.20^a^	13.35 ± 2.75^ab^	12.38 ± 1.15^ab^	15.42 ± 2.88^b^
Weight gain (g)	10.26 ± 1.15^a^	12.62 ± 2.74^ab^	11.87 ± 1.17^ab^	14.69 ± 2.85^b^
SGR (%)	0.97 ± 0.03^a^	1.04 ± 0.09^ab^	1.03 ± 0.06^ab^	1.10 ± 0.07^b^
HSI	0.86 ± 0.37^a^	0.76 ± 0.07^a^	0.87 ± 0.06^a^	0.82 ± 0.07^a^
VSI	1.76 ± 0.40^b^	1.26 ± 0.18^a^	1.75 ± 0.15^b^	1.25 ± 0.21^a^
FCR	2.36 ± 0.23^b^	1.85 ± 0.21^ab^	2.13 ± 0.32^ab^	1.53 ± 0.13^a^
SR (%)	65.80 ± 4.21^b^	76.00 ± 4.32^a^	78.80 ± 4.21^a^	81.60 ± 5.32^a^

BW; body weight, SGR; specific growth rate, VSI; viscerosomatic index; HSI; hepatosomatic index, FCR; feed conversion ratio, SR; survival rate. Values with different alphabetical superscripts in a row differ significantly (p < 0.05) among different probiotics treatments. All values are expressed as mean ± SD.

### 3.3 Histo-morphometry of intestine (first experiment)

**Fig. 1:**
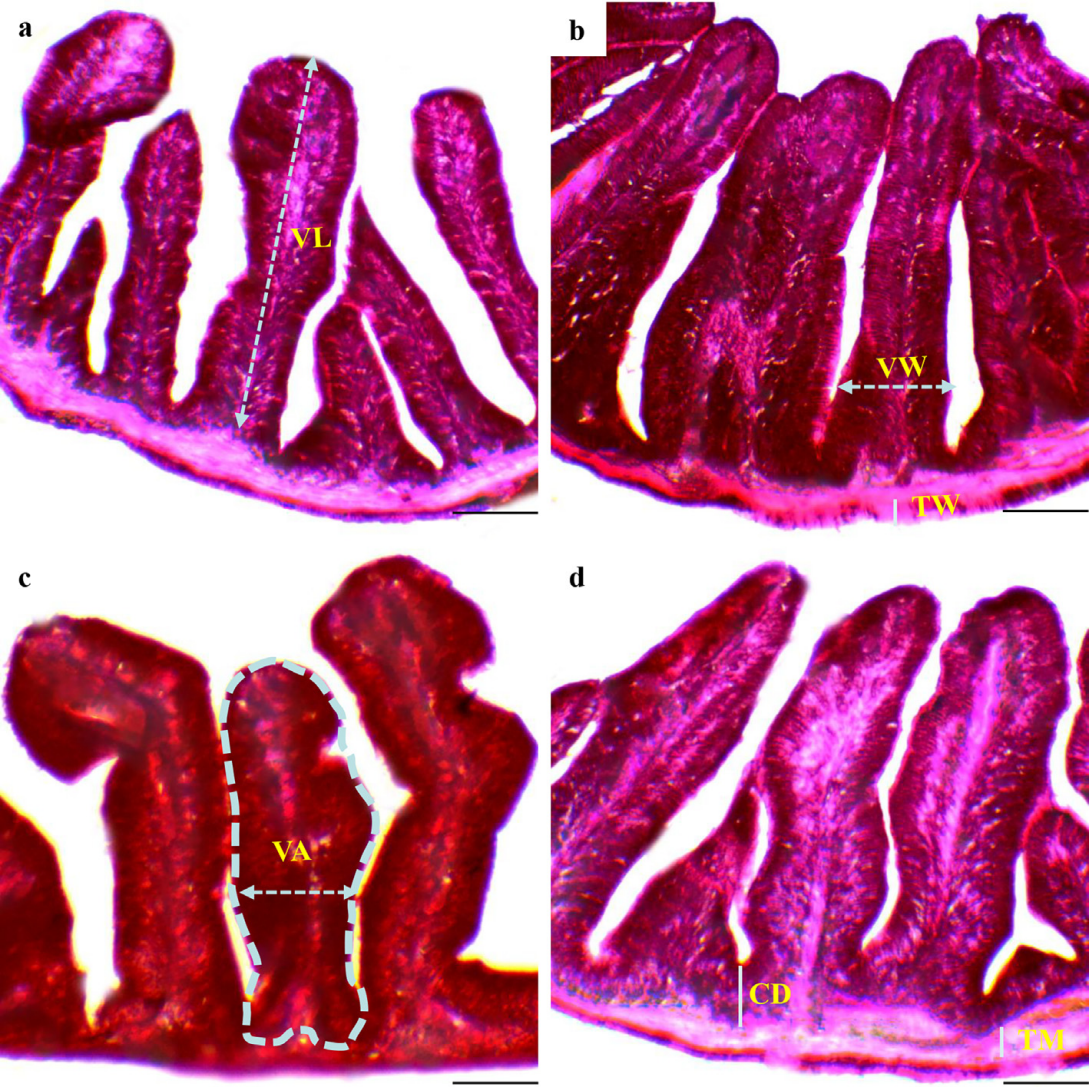
Intestinal histo–morphometric presentation of the Gangetic mystus cultured in aquarium for 8 weeks; (a) control, (b) CP-1, (c) CP-2, (d) Lab dev.; TM; muscular thickness, TW; wall thickness, VW; villus width, VL; villus length, VA; villus area, and CD; crypt depth. Image 40x; scale bar = 100 µm.

**Fig. 2:**
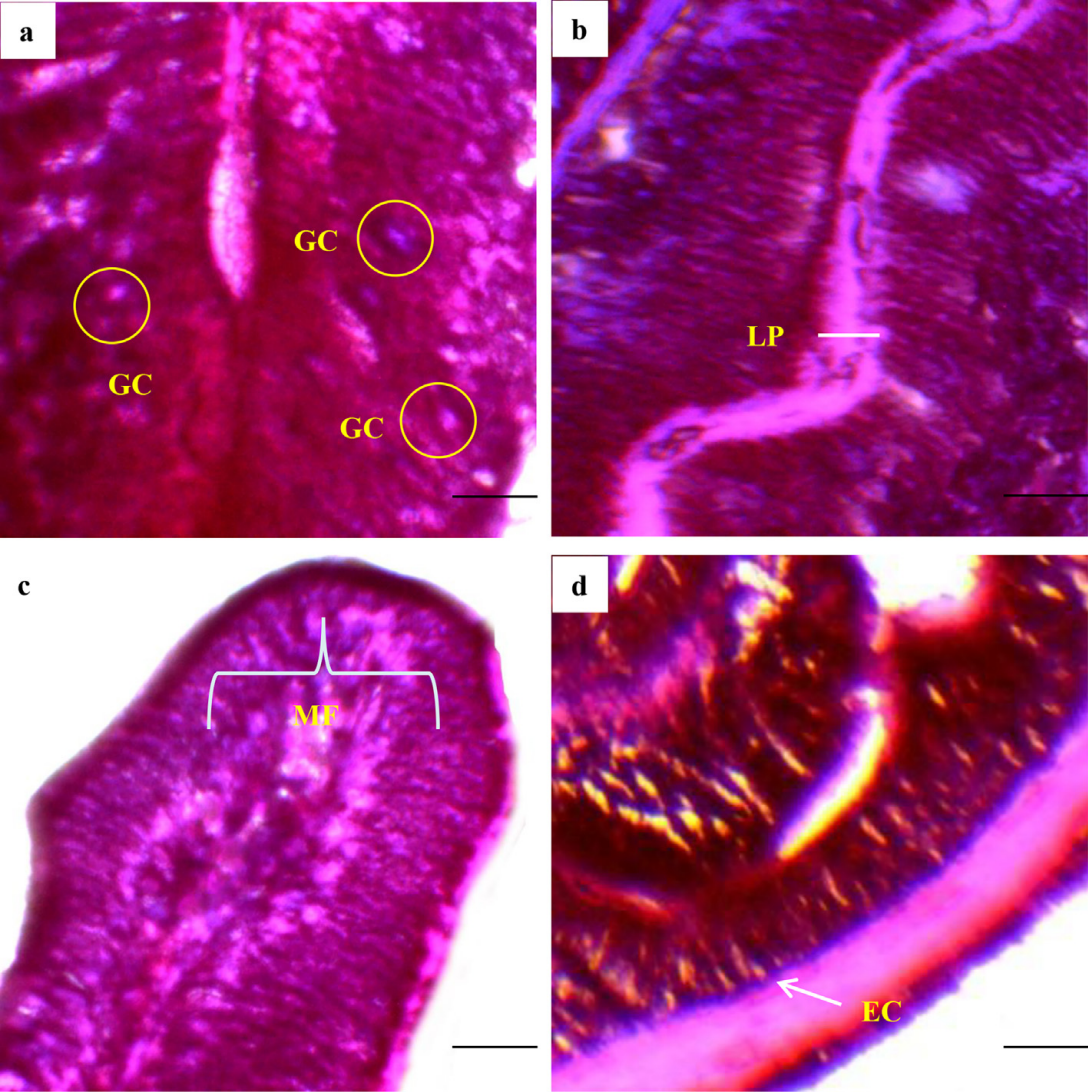
Immune response indicators of Gangetic mystus (*Mystus cavasius*) cultured in aquarium for 8 weeks; GC; goblet cells; MF; mucosal folds, LP; lamina propia, and EC; enterocyte. Image 40x; scale bar = 400 μm.

The observed intestinal histo-morphometry (Fig. 1) from first experiment is illustrated in Table 3. The highest villus length (µm), width (µm) and area (µm^2^) from T3 exhibited significant difference (p < 0.05) compared to others. Crypt depth (µm) and muscular thickness (µm) also recorded highest in T3. The thickness of intestine wall was reported greatest with CP-1 (T1). The findings of immune response indicators (Fig. 2) are shown in Table 4. The maximum abundance in goblet cells, lamina propia width (µm) and fattening of muscular fold (µm) were observed in T3 followed by T2. Enterocyte width (µm) showed no marked variation (p > 0.05) among treatments.

**Table 3:** Findings of intestinal morphometry of the Gangetic mystus (*Mystus cavasius*) reared with probiotics in aquarium for 8 weeks.

Parameters	Probiotics			
	Control	CP-1 (T1)	CP-2 (T2)	Lab dev. (T3)
Villus length (μm)	192.92 ± 9.21^a^	205.50 ± 9.09^ab^	221.50 ± 9.78^ab^	232.00 ± 9.30^b^
Villus width (μm)	29.33 ± 4.13^a^	36.08 ± 5.41^ab^	43.58 ± 8.90^ab^	54.08 ± 8.60^b^
Villus area (mm^2^)	5.59 ± 1.04^a^	7.44 ± 1.33^ab^	9.66 ± 1.90^b^	12.51 ± 1.76^b^
Crypt depth (μm)	9.91 ± 1.59^a^	13.83 ± 2.76^ab^	17.25 ± 4.55^b^	22.91 ± 5.74^b^
Wall thickness (μm)	9.41 ± 3.70^a^	11.67 ± 3.69^b^	7.91 ± 1.74^c^	9.58 ± 2.01^a^
Muscular thickness (μm)	8.08 ± 1.28^a^	13.50 ± 1.89^ab^	16.25 ± 3.49^b^	21.25 ± 3.91^b^

Values with different alphabetical superscripts in a row differ significantly (p < 0.05) among different probiotics treatments. All values are expressed as mean ± SD.

**Table 4:** Immune response features of Gangetic mystus (*Mystus cavasius*) reared in aquarium for 8 weeks.

Parameters	Probiotics			
	Control	CP-1 (T1)	CP-2 (T2)	Lab dev. (T3)
Abundance of goblet cell (GC)	11.33 ± 2.40^a^	15.75 ± 3.87^ab^	20.50 ± 6.92^b^	26.41 ± 5.90^b^
Width of lamina propria (μm)	3.25 ± 0.73^a^	5.67 ± 1.40^ab^	7.83 ± 1.00^b^	11.16 ± 1.24^b^
Fattening of mucosal fold (μm)	13.08 ± 2.55^a^	16.91 ± 2.10^ab^	20.75 ± 2.57^ab^	29.00 ± 6.10^b^
Enterocyte width (μm)	2.83 ± 0.81^a^	2.58 ± 0.50 ^a^	2.58 ± 0.65 ^a^	2.83 ± 0.82 ^a^

Values with different alphabetical superscripts in a row differ significantly (p < 0.05) among different probiotics treatments. All values are expressed as mean ± SD.

### 3.4 Histo-morphometry of liver (second experiment)

**Fig. 3:**
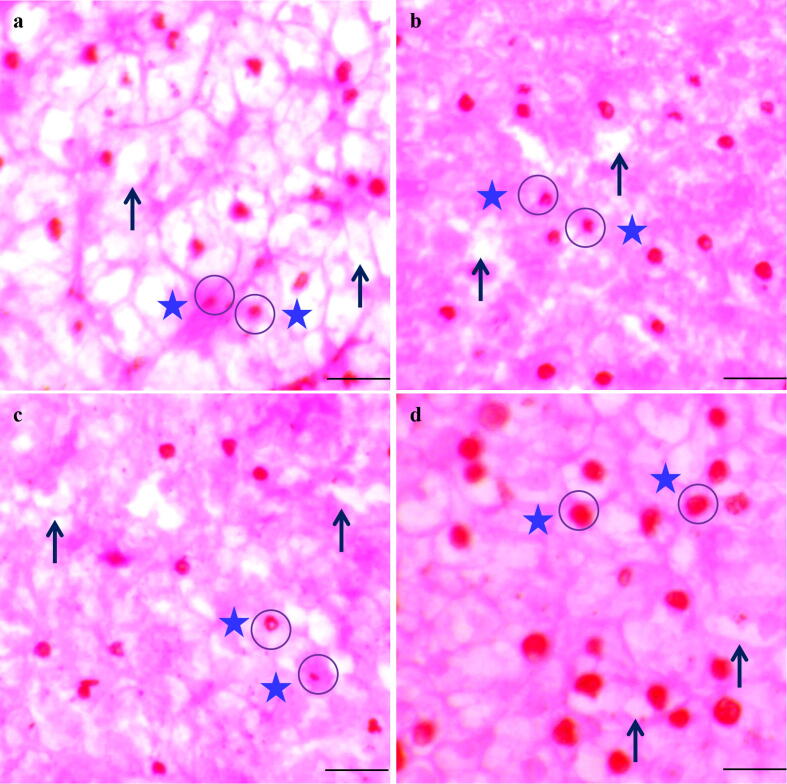
Histo-morphometric changes of liver in Gangetic mystus (*Mystus cavasius*) reared with different probiotics in earthen ponds for 16 weeks; (a) control, (b) CP-1, (c) CP-2, and (d) Lab dev. Arrows shows space among hepatocyte cell and circle shows nucleus shaped. Image 40x; scale bar = 400 µm.

The histo-morphometric changes of liver tissue (Fig. 3) observed in second experiment is depicted in Table 5. The greatest amount of regular shaped nucleus and lowest amount of irregular shaped nucleus were found in T3 that is significantly variant from other treatments (p < 0.05) (Table 5). The least distance between liver tissues (µm) was also observed in T3 that was statistically different (p < 0.05) from control.

**Table 5:** Histo-morphometric changes of liver in the Gangetic mystus (*Mystus cavasius*) reared in earthen ponds for 16 weeks.

Parameters	Probiotics			
	Control	CP-1 (T1)	CP-2 (T2)	Lab dev. (T3)
Irregular shaped nucleus	35.00 ± 3.20^b^	23.00 ± 5.37^ab^	26.25 ± 3.49^ab^	13.75 ± 1.90^a^
Regular shaped nucleus	18.00 ± 2.00^b^	28.50 ± 3.74^ab^	23.50 ± 2.51^ab^	37.25 ± 5.41^a^
Distance of liver tissue (μm)	38.45 ± 2.27^b^	30.30 ± 5.91^ab^	34.55 ± 2.61^b^	18.78 ± 4.20^a^

Values with different alphabetical superscripts in a row differ significantly (p < 0.05) among different probiotics treatments. All values are expressed as mean ± SD.

### 3.5 Blood hemoglobin and glucose (second experiment)

**Fig. 4:**
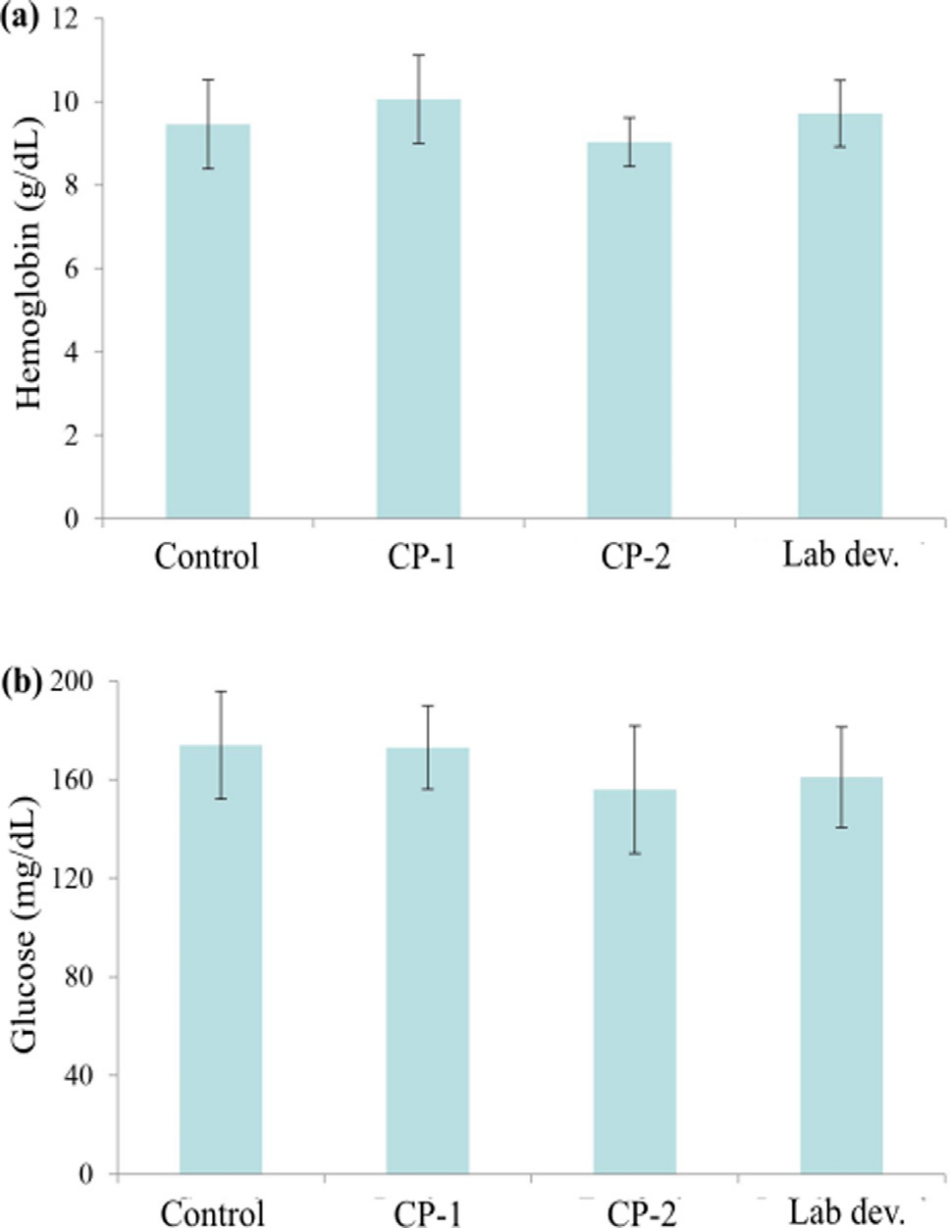
Blood hemoglobin (a) and glucose levels (b) changes in the Gangetic mystus (*Mystus cavasius*) reared for 16 weeks.

The blood hemoglobin (g/dL) and glucose level (mg/dL) observed in Gangetic mystus cultured for 16 weeks are illustrated in Fig. 4. CP-1 (T1) showed highest value for hemoglobin (g/dL) followed by T3 and the lowest was in T1. The level of blood glucose (mg/dL) found minimum in T3, subsequent in T2. The glucose level (mg/dL) showed no marked variation between T2 and T3 (p > 0.05).

### 3.6 Water quality parameters

Water quality parameters were estimated for both of the experiments and are presented in Table 6. The mean temperature ranged from 31.17 °C to 31.67 °C and 32.14 °C to 32.24 °C; pH ranged from 8.54 to 8.79 and 8.60 to 8.64; and lowest average of ammonia was observed in T3 for first and second experiment, respectively.

**Table 6:** Water quality parameters of the Gangetic mystus (*Mystus cavasius*) reared with different probiotics (All values are expressed as mean ± SD).

Experiments	Parameters	Probiotics			
		Control	CP-1 (T1)	CP-2 (T2)	Lab dev. (T3)
First	Temperature (°C)	31.50 ± 0.91	31.17 ± 0.84	31.22 ± 0.91	31.67 ± 0.76
	DO (mg/L)	7.85 ± 0.31	7.90 ± 0.38	7.82 ± 0.33	7.95 ± 0.29
	pH	8.70 ± 0.16	8.79 ± 0.05	8.54 ± 0.45	8.64 ± 0.15
	NH_3_ (mg/L)	0.29 ± 0.45	0.38 ± 0.33	0.33 ± 0.14	0.25 ± 0.25
Second	Temperature (°C)	32.14 ± 1.89	32.24 ± 2.00	32.15 ± 1.55	32.21 ± 2.15
	DO (mg/L)	8.60 ± 2.05	7.91 ± 1.05	8.90 ± 0.98	7.48 ± 1.78
	pH	8.60 ± 0.31	8.67 ± 0.27	8.64 ± 0.04	8.65 ± 0.31
	NH_3_ (mg/L)	0.37 ± 0.37	0.34 ± 0.20	0.32 ± 0.05	0.27 ± 0.14

### 3.7 Principal component analysis (PCA)

**Fig. 5:**
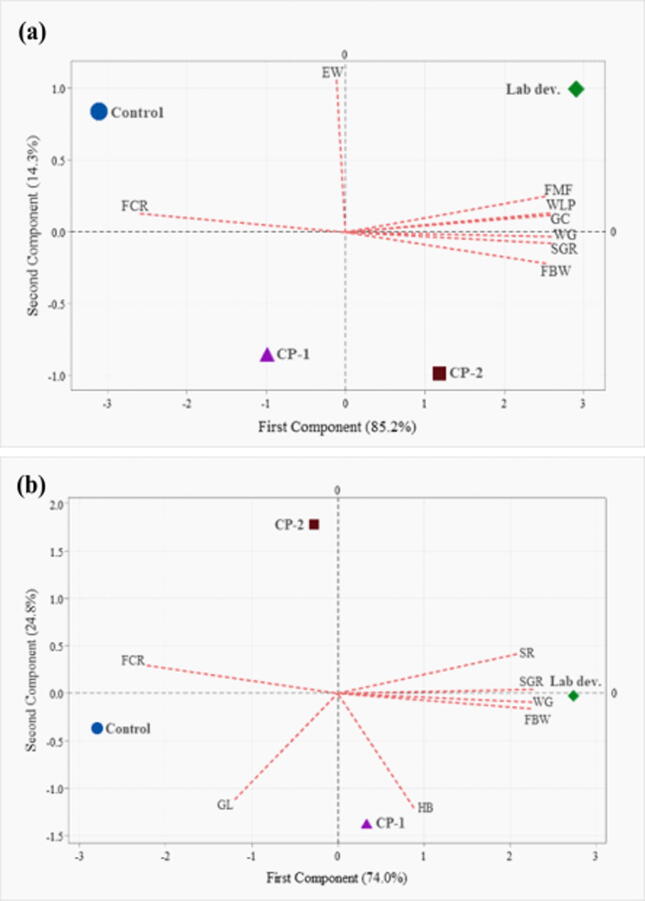
Biplot of growth, feed utilization and immune response of Gangetic mystus cultured in aquaria for 8 weeks (a) and Biplot of growth, feed utilization, survival and hematological parameters of Gangetic mystus cultured in earthen ponds for 16 weeks (b). FBW; final body weight, WG; weight gain, SGR; specific growth rate, VSI; viscerosomatic index; HSI; hepatosomatic index, FCR; feed conversion ratio, SR; survival rate; GC; goblet cells; FMF; fattening of mucosal folds, WLP; width of lamina propia, EW; enterocyte width, HB; hemoglobin, and GL; glucose.

Biplot of growth, feed utilization and immune response of Gangetic mystus cultured in aquaria is shown in Fig. 5a. The first component (85.2%) and second component (14.3%) represented the total 99.5% of the cumulative variance. The graph exhibited that Lab dev. probiotic (T3) brought positive change in terms of growth, feed conversion efficiency and immune response followed by CP-2. The parameters of growth (final body weight, FBW; weight gain, WG; specific growth rate, SGR) and immune response (abundance of goblet cell, GC; width of lamina propia, WLP; fattening of mucosal fold, FMF) are positively correlated with each other and poor FCR separates control from other treatments. Biplot of growth, feed utilization, survival and hematological parameters of Gangetic mystus cultured in earthen ponds is shown in Fig. 5b. Again, Lab dev. probiotic anticipated better performance in survival (SR) and growth factors (FBW, WG and SGR). The cumulative percentage of variance was 98.8% for first two components. Blood glucose and FCR separates control from CP-1 (T1) and CP-2 (T2), respectively.

## 4 Discussion

This study was purposed to assess the effects of two selective commercial and a laboratory developed probiotics on growth, feed conversion efficiency, survival, immune response and hematological parameters of Gangetic mystus (*Mystus cavasius*) in aquaria (8 weeks) and earthen ponds (16 weeks). The administration of probiotics, in this study, exhibited substantial improvement in growth performance, feed utilization, survival, histo-morphometry of intestine, liver as well as hematological parameters in Gangetic mystus.

Likewise this study, numerous scientific literatures documented that the inoculation of beneficial bacteria either in water or in feed stimulated growth performance, nutrient digestibility (indicated by low FCR) and survival for different fishes and shrimps such as, *Bacillus subtilis* increased weight gain and feed utilization for grouper (*Epinephelus coioides*) in 28 day culture (Liu et al., 2017); incorporation of *B. cereus* and *B. thuringiensis* enhanced final weight and feed conversion efficiency in Asian seabass, *Lates calcarifer* (Ghanei-Motlagh et al., 2021); mixture of *Bacillus* spp., and *Lactobacillus* spp. improved weight gain and SGR in Nile tilapia (*Oreochromis niloticus*), and carp (*Cirrhinus cirrhosus*) (Apún-Molina et al., 2009, Hossain et al., 2022a, Hossain et al., 2022b, Ramos et al., 2017); *B. coagulans* and *Rhodpseudomonas palustris* together resulted better growth performance and low FCR for Nile tilapia (Zhou et al., 2010); *Bacillus* sp., *Vibrio* sp., and *Lactobacillus plantarum* increased survival along with improved weigh gain and SGR in freshwater shrimp (*Macrobrachium rosenbergii*) (Mujeeb Rahiman et al., 2010); *Lactobacillus acidophilus* supplement positively reflected in growth and FCR for African catfish, *Clarias gariepinus* (Al-Dohail et al., 2011); usage of dietary probiotic mixture of *B. subtilis*, *B. megaterium*, and *B. licheniformis* increased the net production, feed conversion, survivability of channel catfish (*Ictalurus punctata*) (Queiroz and Boyd, 1998); yeast (*Saccharomyces cerevisiae*) and *Acinobacter* ensured better growth and feed utilization in striped catfish, *Pangasianodon hypophthalmus* and Bighead catfish, *Clarias macrocephalus*; respectively (Boonanuntanasarn et al., 2019, Bunnoy et al., 2019). In general, feed conversion efficiency and wellbeing of fish are reliant on the enzymatic activity, nutrient digestibility and absorption, similarly, better digestion and nutrient assimilation results better performance in growth. The digestion and nutrient absorption are believed to be accelerated by probiotic usage as bacterial colony has potency in stimulating hydrolytic extracellular digestive enzymes like amylase, chitinase, protease and lipase (Balcázar et al., 2006a; El-Saadony et al., 2021a; Hai, 2015). Besides, bacterial protease enzyme enhances the digestion rate through breakdown complex protein content with the release of essential amino acids that are considered as nutrient supplement (Ray et al., 2012). Moreover, probiotics can be a vital source of growth stimulators like essential fatty acids, cobalamin (B12), biotin (B7) and vitamin K (Balcázar et al., 2006, El-Saadony et al., 2021). In a nutshell, bacterial proliferation increases metabolism and triggers appetite that ensures proper digestion, utilization of feed (Irianto and Austin, 2002) and results improved growth and FCR.

Histological observation of intestine is helpful to understand the structure and functioning of healthy gut. This study revealed that the administration of probiotic positively enhanced various histo-morphometric dimensions of intestine such as villi length, width, area, crypt depth and thickness of intestine muscle (Table 3). The increased villi dimensions like length and thickness are indicators of improved intestinal surface area that ultimately induces the performance of intestine in terms of digestion and absorption (Haque et al., 2021). Apart from digestion and absorption, intestine plays significant role in generating immune response and acts as first-line defense against infection (Secombes and Ellis, 2012). Here, immune response indicators such as amount of goblet cells, lamina propia width and thickness of mucosal fold are observed to be escalated with the application of probiotics. Studies found that probiotics as feed additives increase absorption surface area and number of goblet cells in Nile tilapia (Elsabagh et al., 2018, Standen et al., 2016). However, goblets safeguard the intestine through secreting mucus (mucin) along with other immune stimulating substances that saves intestine from being dehydrated as well as its lubricating properties ensure the disintegration of harmful pathogens (Cain et al., 1996, McGuckin et al., 2011). The fattening of intestinal mucosa fold provides more space in order to absorb more nutrient that has direct impact on growth and immunity (Islam et al., 2021, Jahan et al., 2021). El Euony et al., (2020) reported that probiotic (*B. subtilis*) in fed boosted the immunity of African catfish (*Clarias gariepinus*). Histo-morphometric features of liver in present study showed that usages of probiotic positively increased the regular shaped nucleus with remarkable reduction of irregular nucleus and ensured least intracellular distance between hepatic tissues (Table 5). These findings indicated healthier morphological structure and functioning of liver due to probiotic administration. According to Ruiz et al., (2020), probiotic helps to regenerate hepatocytes and fosters better functioning. In addition, excess accumulated fat in liver can be reduced through probiotics usage (Hossain et al., 2022a).

In this experiment, the hemoglobin concentration for Gangetic mystus varied between 9.0 and 10.0 g/dl (Fig. 4). Mlay et al. (2007) observed that the average hemoglobin concentration of African catfish was 9.7 g/dl and Nile tilapia was 9.2 g/dl which are comparable with present study. Dahiya et al. (2012) also enlisted hemoglobin level of walking catfish (*Clarias batracus*) for different probiotic treatments. The hemoglobin concentration usually higher in younger age, decrease slightly in juvenile phase and then become steady (Mlay et al., 2007). Blood glucose is one of the core energy sources that plays dynamic role in cell metabolism whereas, hyperglycemia is an indicator of environmental stress as the homeostatic condition of fish easily disturbed by the instantaneous changes in environments (Malini et al., 2018, Shahjahan et al., 2022). Increased blood glucose might occur due to physiological stress that excites hypothalamus of central nervous system and stimulate the release of cortisol and catecholamine from adrenal glands (Beyea et al., 2005). Besides, glucose concentration might vary depending on fish species, age, habitat and environmental factors as well (Malini et al., 2018). In our study, the mean glucose level ranged from 158.0 to 172.0. Patriche, (2009) observed that the normal blood glucose of cyprinids ranges from 40 to 90 mg/dl and Hossain et al. (2022a) reported 118.8–144.0 mg/dl for mrigal (*Cirrhinus cirrhosus*) reared in probiotic. However, to the best of our knowledge, the level of glucose for catfish blood due to probiotic administration is not well established yet.

Although Lab dev. probiotic showed best overall performance, the commercial probiotics CP-2 exhibited positive results in aquaria where less environmental factors influenced the culture; CP-1 imparted comparatively good result especially in pond interacting with more environmental factors (Fig. 5). The authors believe that the reasons behind the effectivity of Lab dev. probiotics might be its bacterial composition. The commercial probiotics used in this experiment contains multi-strains of *Bacillus*, and *Aspergillus* (CP-1) and multi-species of *Bacillus*, *Rhodococcus*, *Rhodobacter*, *Nitrosomonus*, *Nitrobacter* (CP-2) whereas Lab dev. probiotic is comprised of multi-strains of *Bacillus* spp., and *Lactobacillus* spp. The best performances was observed in Lab dev. probiotic treatment in terms of growth, feed utilization, histo-morphometry and immunity and hematological parameters implied that *Bacillus* spp. along with *Lactobacillus* spp. has profound significance in the rearing of Gangetic mystus. However, further investigations could be carried out on the enzymatic activities, disease resistance, and gene expression due to probiotic administration. In addition, determination of which specific bacterial strains’ play most important role for better growth and immunity in fishes to be another focus of future researches.

## Declaration of Competing Interest

The authors declare that they have no known competing financial interests or personal relationships that could have appeared to influence the work reported in this paper.
